# Sigmoido-rectal intussusception anastomosis in the Altemeier procedure for complete rectal prolapse: preliminary results of a new technique

**DOI:** 10.3389/fsurg.2024.1340500

**Published:** 2024-02-05

**Authors:** Benjun Wang, Weiwei Han, Yuze Zhai, Renjie Shi

**Affiliations:** ^1^ First Clinical Medical College, Nanjing University of Chinese Medicine, Nanjing, China; ^2^ Department of Anorectal Surgery, Affiliated Hospital of Shandong University of Traditional Chinese Medicine, Jinan, Shandong, China; ^3^ First Clinical Medical College, Shandong University of Traditional Chinese Medicine, Jinan, Shandong, China; ^4^ Department of Anorectal Surgery, Affiliated Hospital of Nanjing University of Chinese Medicine, Nanjing, China; ^5^ Department of Anorectal Surgery, Jiangsu Province Hospital of Traditional Chinese Medicine, Nanjing, China

**Keywords:** complete rectal prolapse, Altemeier procedure, mortality, recurrence, complications

## Abstract

**Purpose:**

Our research introduces an innovative surgical approach, combining the Altemeier Procedure with Sigmoido-rectal Intussusception Anastomosis, effectively reducing recurrence, minimizing complications, and improving postoperative anal function in rectal prolapse patients.

**Materials and methods:**

This retrospective study, conducted at tertiary referral hospitals including Shandong University of Traditional Chinese Medicine's Affiliated Hospital, Linyi People's Hospital, and Pingyi People's Hospital, examined data from patients undergoing conventional Altemeier surgery or Altemeier combined with Sigmoido-rectal Intussusception Anastomosis. Analyzing hospitalization and follow-up data from January 2009 to December 2022, the study focused on prolapse recurrence, complications, and anal function as primary outcome indicators across these three study centers.

**Results:**

In the study, both groups had an average follow-up of (12.5 ± 2.41) months, and only two traditional group patients experienced mortality. Recurrence rates significantly differed, with 26.47% in the traditional group and 1.54% in the modified group (*P *< 0.001). The modified group showed no perioperative anastomotic dehiscence, contrasting with a 13.24% occurrence in the conventional group (*P *= 0.003). Primary complications in the modified group included anastomotic hemorrhage, with rates of 17.65% and 6.15% in the traditional and modified groups, respectively (*P *= 0.077). At 12 months postoperatively, both groups improved in anal manometry parameters and the Wexner anal incontinence score. Resting pressure was significantly lower in the traditional group (32.50 ± 1.76 mmHg) than the modified group (33.24 ± 2.06 mmHg) (*P *= 0.027), while the extrusion pressure was higher in the modified group (64.78 ± 1.55 mmHg) than the traditional group (62.85 ± 2.30 mmHg) (*P* < 0.001). The Wexner anal incontinence score was significantly lower in the modified group (2.69 ± 1.65) than the traditional group (3.69 ± 1.58, *P *= 0.001).

**Conclusion:**

This retrospective study affirms that adding Sigmoido-rectal Intussusception Anastomosis to the Altemeier procedure reduces recurrence and complications. While both approaches enhance postoperative anal function in complete rectal prolapse patients, the combined method, particularly with Sigmoido-rectal Intussusception Anastomosis, proves more effective.

## Introduction

Complete rectal prolapse (CRP) is a benign pelvic floor disorder with a prevalence of 2.5 per 100,000 in the general population, most commonly observed in the elderly and children (1). Surgery is the primary and most effective treatment for complete rectal prolapse (CRP), with the goal of restoring pelvic anatomy, correcting the prolapse, and improving defecation. There are over 130 surgical procedures available for the treatment of CRP, primarily categorized into abdominal and perineal surgery (2).

Conventional wisdom suggests that postoperative recurrence rates after transabdominal surgery for CRP are approximately one-fourth of those seen with transperineal surgery, often yielding better postoperative anal function outcomes. However, a systematic evaluation of the Cochrane database found no significant difference in the recurrence rate of rectal prolapse between transabdominal and perineal surgery. Furthermore, transperitoneal surgery requires a higher level of patient fundamentals (3) and is associated with a higher complication rate, including pelvic adhesions, incisional hernia, sexual dysfunction, and anastomotic issues within 30 days, with complication rates ranging from 0 to 20%.

On the contrary, perineal surgery may be recommended for patients with a poor general condition, where the higher risk of recurrence (4) can be balanced by the patient's shorter life expectancy and the potential for repeating the procedure. Consequently, the Altemeier procedure is a representative technique in transperineal surgery, commonly used for elderly patients, those with significant surgical risks, men, and in cases of acute strangulated prolapse (5).

The recurrence rate of the Altemeier procedure is a topic of debate, with some reports indicating a maximum of 29%. This variability may be related to the patient's physical condition (6, 7) and the surgeon's experience.

Current research indicates that “intussusception” in the mid-rectum is the primary pathogenesis of rectal prolapse. While surgery can address identified anatomical defects in CRP, the presence of “intussusception” may contribute to both pathogenesis and recurrence (8). Our study team has synthesized the “intussusception” theory, suggesting that pathological dilatation of the distal rectum is another anatomical defect in CRP. This defect might serve as the “trigger point” for recurrence following conventional Altemeier's procedure.

On one hand, the significant change in the diameter of the distal pathologically dilated rectal lumen compared to the diameter of the sigmoid colon lumen (at the proximal anastomosis) makes it challenging to withstand excessive intra-abdominal pressure after conventional Altemeier's surgery. This can lead to the re-projection of the sigmoid colon outside the anus, resembling a type of “sliding hernia”, ultimately resulting in the recurrence of rectal prolapse.

On the other hand, pathological dilatation of the distal rectum and repeated prolapse of rectal prolapse can lead to damage and degeneration of intestinal muscle fibers in the intestinal wall, resulting in a decreased postoperative healing ability and local complications, such as anastomotic tears, following the traditional Altemeier procedure.

In response, we performed a combined Sigmoido-rectal intussusception anastomosis alongside the Altemeier procedure to address these anatomical defects. Our goal was to reduce the postoperative recurrence and complication rates following traditional Altemeier surgery while enhancing postoperative anal function. In this article, we present our preliminary results, focusing on safety and efficacy, with short-term recurrence rate, complication rate, and anal function as our primary outcome indicators.

## Material and methods

### Study population and inclusion criteria

A retrospective analysis was conducted on the clinical records of 144 patients diagnosed with Complete rectal prolapse (CRP) who underwent either the traditional Altemeier procedure or the Altemeier procedure combined with Sigmoido-rectal intussusception anastomosis. This study spanned from January 2019 to December 2022 and was carried out at three reputable medical institutions: the Affiliated Hospital of Shandong University of Traditional Chinese Medicine, Linyi People's Hospital of Shandong Province, and Pingyi People's Hospital of Shandong Province.

Data collection for this analysis was facilitated through the utilization of a meticulously designed data sheet, which was employed both during the patients' hospitalization and at subsequent follow-up assessments.

Preoperative Evaluations:

1. Patient Assessment: Prior to surgery, both patient groups underwent a comprehensive series of preoperative tests, which included the following:
  a) Wexner Anal Incontinence Score: Conducted on the day before admission, the Wexner score, ranging from 0 (indicating full continence) to 20 (indicating complete incontinence) (9), was used to evaluate anal incontinence. In addition, patients' symptoms, encompassing dyschezia, tenesmus, anorectal pain, and rectal bleeding, were systematically recorded.
  b) Anal Manometry: Employed to measure specific parameters, such as:
    - Resting Pressure in the Anal Canal: Calculated as the average pressure in the recording channel with the highest pressure at the end of the resting period.
    - Squeeze Pressure: Determined as the average maximum pressure in the recording channel with the highest pressure during the squeeze.
2. Specialized Examination: Preceding the surgical procedures, a specialized examination of the perineal area, rectum, and vagina was conducted with the aim of assessing various factors, including:
  - The maximum degree of rectal prolapse.
  - The effectiveness of casual muscle contractions.
  - The presence of any other genital prolapse.
3. Colonoscopy: Colonoscopy was systematically performed to rule out the presence of any associated colorectal diseases.

Inclusion and Exclusion Criteria:

a) Inclusion Criteria:
  - Rectal Procidentia: Patients included in this study exhibited rectal procidentia exceeding 5 cm.
  - Fecal Incontinence: Patients with a fecal incontinence score exceeding1were considered for inclusion.
  - Absence of Absolute Contraindications: Only patients without absolute contraindications to surgery were included.
b) Exclusion Criteria:
  - Rectal Procidentia: Patients with rectal procidentia measuring less than 5 cm were excluded.
  - Absolute Contraindications: Exclusion criteria included the presence of absolute contraindications to surgery, such as inflammatory bowel disease, cancer, mental disorders, and patients with contraindications to anesthesia.
  - Coexistence of Other Organ Prolapse: Patients with other organ prolapse were also excluded from the study.

### Surgical technique

#### Preoperative management

As part of our standard protocol, routine antibiotic prophylaxis was administered in the form of oral laxatives for bowel cleansing and oral ornidazole dispersible tablets (500 mg) three days prior to the surgery. The surgical procedures were consistently performed by the same experienced surgeon with the patient in the lithotomy position.

The Altemeier procedure, as referenced in prior literature (10), was employed in this study.

**Figure 1:**
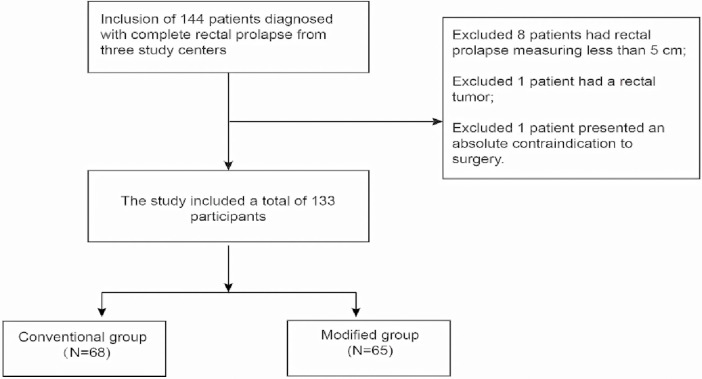
Flowchart of this retrospective study.

**Figure 2:**
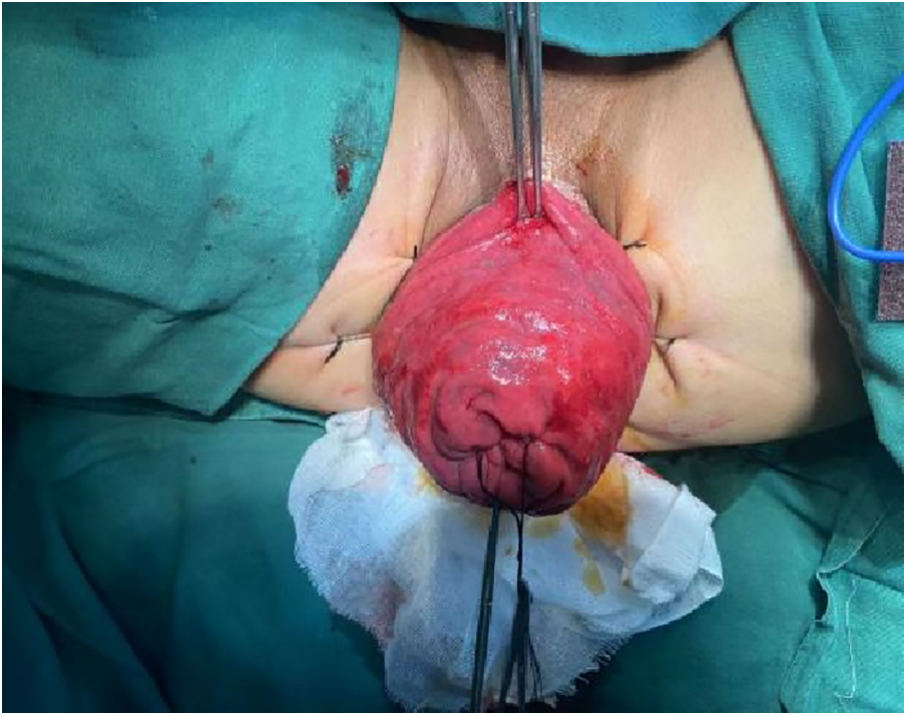
Six equidistant stitches were placed in the perianal area. The prolapsed bowel was delicately extracted using tissue forceps.

**Figure 3:**
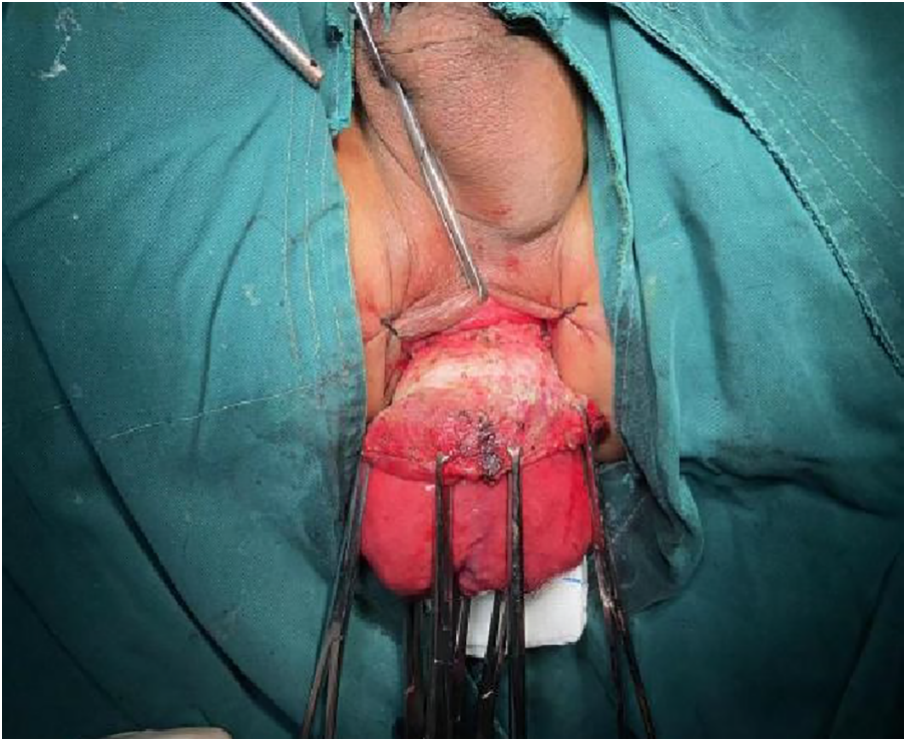
Marked a pre-excision line located 1–1.5 cm proximal to the dentate line, with a focus on preserving the anal cushion.

**Figure 4:**
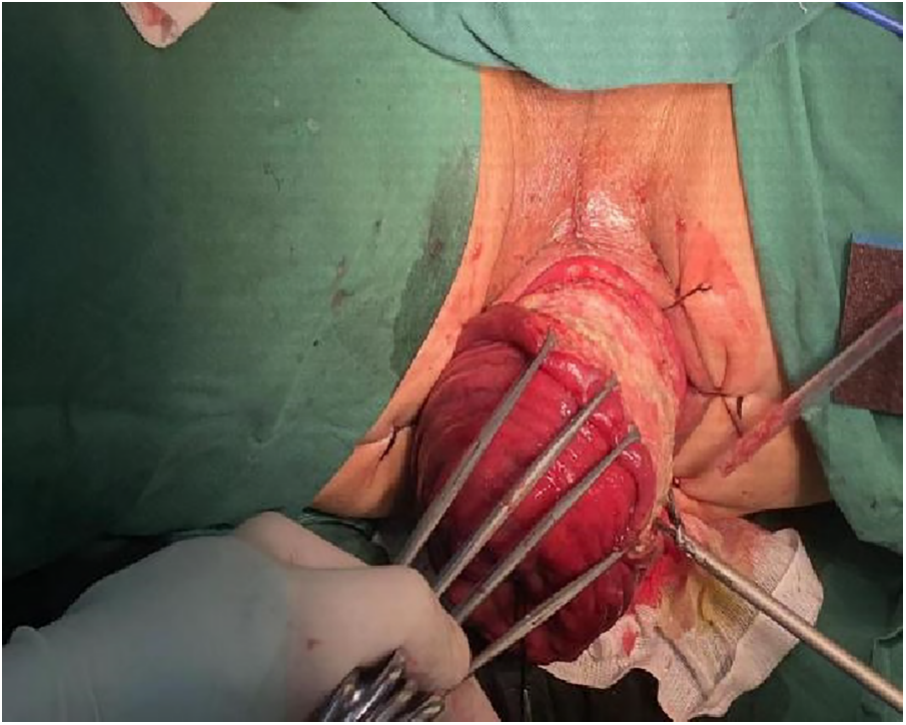
Peeling of the rectal mucosa while preserving a 2–3 cm section of the rectal muscular sheath.

**Figure 5:**
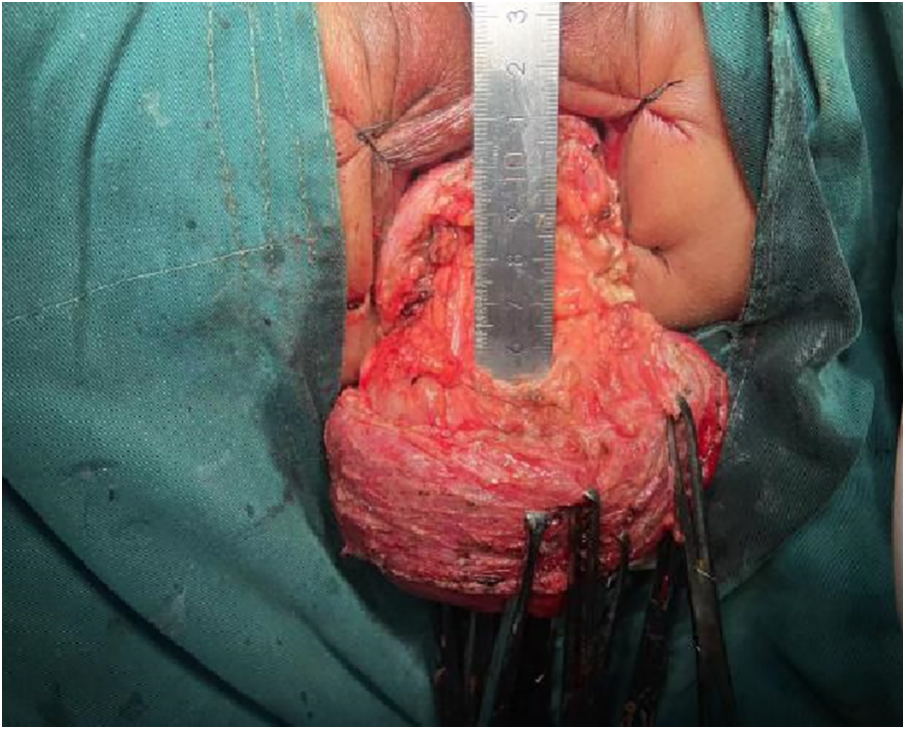
Incisions extended into the depth of the Douglas’ notch, with lateral expansion to ensure thorough liberation of the prolapsed bowel and rectal mesentery.

**Figure 6:**
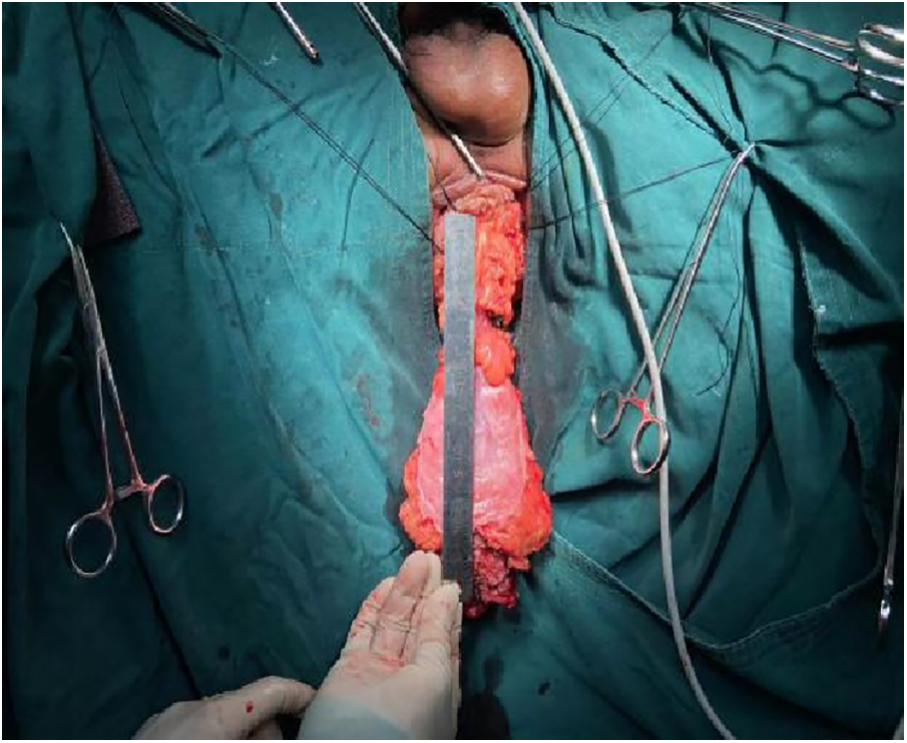
Incisions extended into the depth of the Douglas’ notch, with lateral expansion to ensure thorough liberation of the prolapsed bowel and rectal mesentery.

**Figure 7:**
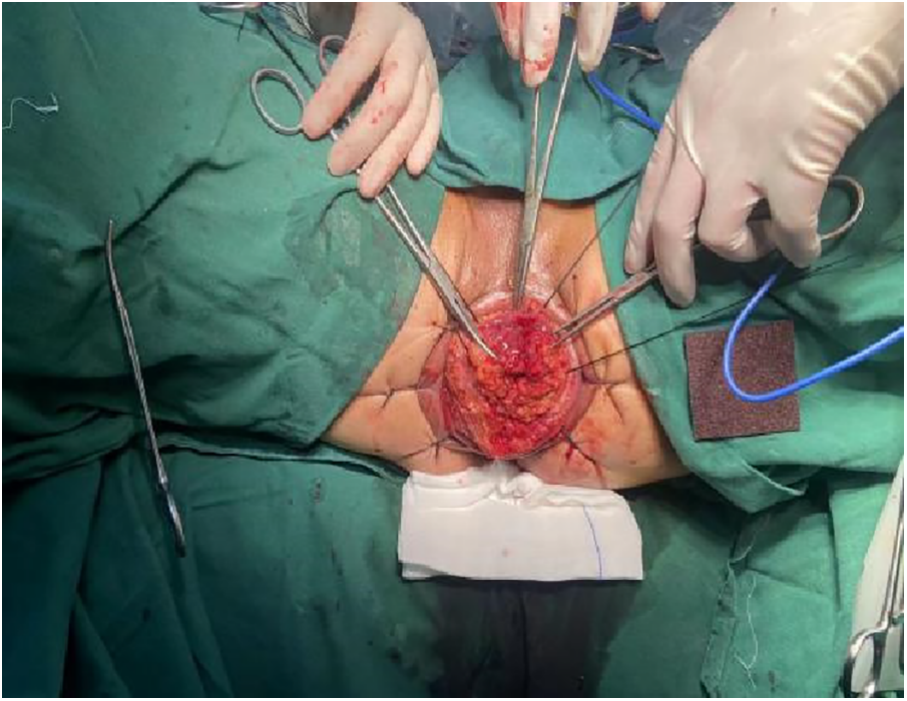
The proximal 3–5 cm from the closed pelvic floor was designated as the pre-excision line for the medial intestines, facilitating the removal of the prolapsed bowel.

**Figure 8:**
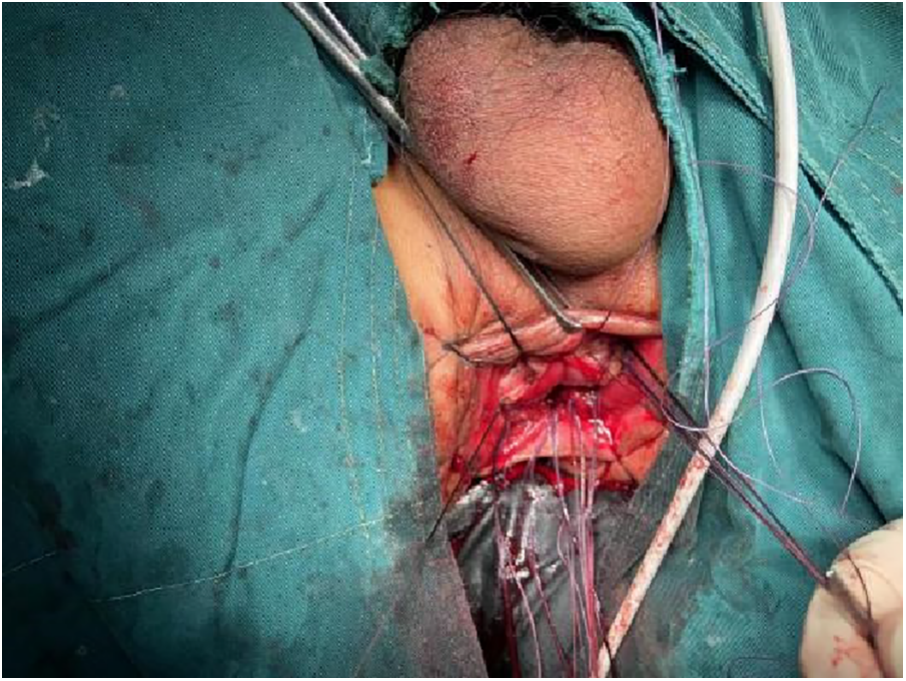
Pulling the proximal colon into the myenteric sheath: using 3-0 absorbable thread, intermittent sutures were applied to create an anastomosis between the tip of the myenteric sheath of the distal preserved portion of the rectum and the serosa layer of the proximal end of the colon, which was pulled into the sheath. This ensured that the proximal end of the colon was nestled into the distal muscle sheath by approximately 2–3 cm.

**Figure 9:**
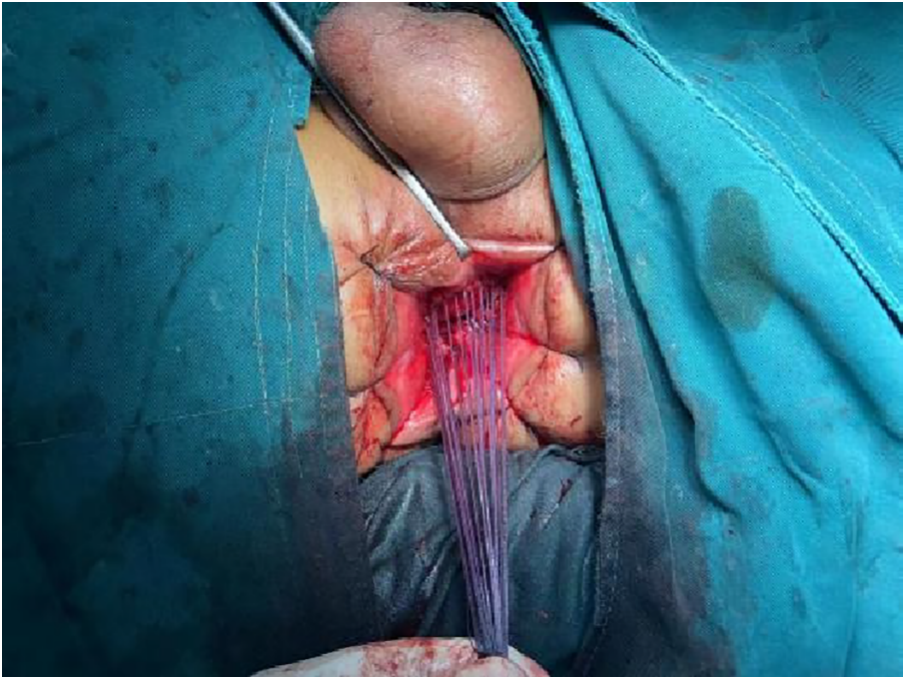
Mucosal and muscular layer anastomosis: the muscular and mucosal layers of the incision in the distal rectum were anastomosed with intermittent sutures to the full thickness of the proximal colon. Particular attention was given to ensure tight alignment of the mucosal layers, thereby situating the anastomosis within the muscular sheath for protection.

The surgical procedure employed in this study involved Altemeier's technique combined with Sigmoido-rectal intussusception anastomosis. The procedure consisted of several key steps:

1. Exposure and Bowel Extraction: To provide adequate surgical exposure, six equidistant stitches were placed in the perianal area. The prolapsed bowel was delicately extracted using tissue forceps (Figure 1).
2. Rectal Mucosal Dissection: In contrast to traditional surgery, we marked a pre-excision line located 1–1.5 cm proximal to the dentate line, with a focus on preserving the anal cushion (Figure 2). An ultrasonic scalpel was used for annular dissection of the mucosal layer down to the submucosa. This was followed by toroidal peeling of the rectal mucosa while preserving a 2–3 cm section of the rectal muscular sheath (Figure 3). Subsequently, incisions were made through the rectal muscular layer, serosa layer, and pelvic floor peritoneum along the anterior wall of the rectum, layer by layer. This process extended into the depth of the Douglas' notch, with lateral expansion to ensure thorough liberation of the prolapsed bowel and rectal mesentery (Figures 4, 5). Stripping was performed up to approximately 2–3 cm proximal to the cut edge of the preset anastomosis. Preservation of the rectal mesentery was crucial to maintain blood supply to the retained bowel segments. Additionally, a segment of the pelvic floor peritoneum, approximately 2–3 cm, was conserved and subsequently closed with sutures. This preserved pelvic floor peritoneum was employed to reinforce the anterior wall of the rectum in a tension-free manner. The proximal 3–5 cm from the closed pelvic floor was designated as the pre-excision line for the medial intestines, facilitating the removal of the prolapsed bowel (Figure 6). Finally, anorectal muscle was posteriorly folded within the rectum to perform anorectal myoplasty.
3. Sigmoido-Rectal Intussusception Anastomosis Innovation: Specific modalities of this innovation included:
  a) Pulling the Proximal Colon into the Myenteric Sheath: Using 3-0 absorbable thread, intermittent sutures were applied to create an anastomosis between the tip of the myenteric sheath of the distal preserved portion of the rectum and the serosa layer of the proximal end of the colon, which was pulled into the sheath. This ensured that the proximal end of the colon was nestled into the distal muscle sheath by approximately 2–3 cm (Figure 7).
  b) Mucosal and Muscular Layer Anastomosis: The muscular and mucosal layers of the incision in the distal rectum were anastomosed with intermittent sutures to the full thickness of the proximal colon. Particular attention was given to ensure tight alignment of the mucosal layers, thereby situating the anastomosis within the muscular sheath for protection (Figure 8, 9).

Notably, no prophylactic ileostomy was performed in any of the patients.

#### Postoperative management and follow-up indicators

The duration of surgery and the amount of blood loss were meticulously recorded for each case, and all surgical specimens underwent comprehensive measurement and examination. Following the surgical procedures, patients were placed on postoperative antibiotic regimens, and their anal region was diligently monitored twice daily for any indications of bleeding or signs of infection.

Postoperatively, the introduction of food was initiated after the first defecation, with a gradual transition from a liquid diet to a regular diet. Patients experiencing constipation were administered oral lactulose or polyethylene glycol to manage the condition.

A postoperative schedule was established for patients, including clinical examinations at 2 weeks and at 3, 6, and 12 months following the surgery. Subsequently, patients underwent annual check-ups for a period of 5 years, during which even small, asymptomatic recurrences of CRPs were carefully monitored.

**Figure 10:**
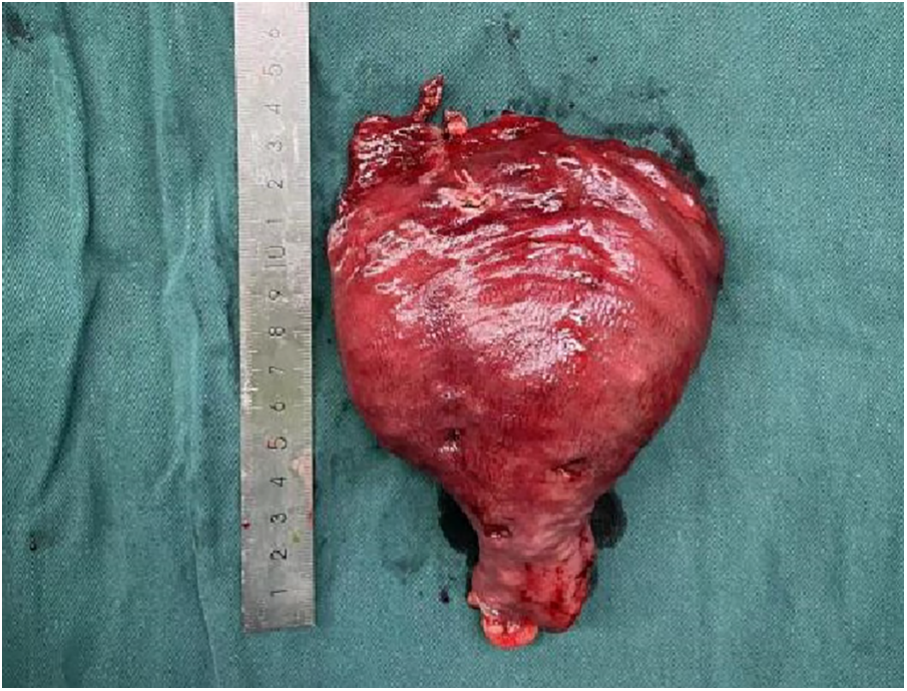
Distal pathologically dilated rectum.

The distal pathologically dilated rectum was identified using a surgical measuring tape (Figure 10).

The collected data encompassed various parameters, including operative time, length of resected bowel, blood loss, duration of hospitalization, early and late complications, anal manometry results, recurrence rates, and mortality.

### Calculation and statistical analysis

Statistical analyses were performed using a range of methods, including Chi-Square, Fisher's exact test, *t*-tests for unpaired data, and the Mann–Whitney *U*-test as deemed appropriate for the given data. Regression analysis was also employed.

Significance was determined at the 95% confidence level, with a threshold set at *P *< 0.05 to establish statistical significance.

## Results

Following adherence to the inclusion and exclusion criteria, a total of 10 patients were excluded from this study. Of these exclusions, 8 patients had rectal prolapse measuring less than 5 cm, 1 patient had a rectal tumor, and 1 patient presented an absolute contraindication to surgery. Consequently, the study included a total of 133 participants, all of whom were undergoing rectal prolapse surgery for the first time. All participants had a rectal prolapse length exceeding 5 cm, and none had any absolute contraindications to surgery.

Among the participants, 68 were allocated to the Conventional group, while the remaining 65 were assigned to the Modified group. Each patient provided written informed consent and underwent the surgical procedures carried out by the same surgeon, BJ, using the aforementioned surgical techniques. The study received approval from the ethical committees of the Affiliated Hospital of Shandong University of Traditional Chinese Medicine, Linyi Municipal People's Hospital, and the Linli Committee of Pingyi County People's Hospital.

For a visual representation of the study's flow, please refer to Flowchart in the accompanying.

### Baseline characteristics of the study population

Throughout the study period, a total of 68 patients (comprising 57.35% males) underwent conventional Altemeier surgery, constituting the conventional group. The mean age of this group was 40.87 years, with a standard deviation of 18.96 years.

Similarly, 65 patients (52.31% males) underwent the modified Altemeier surgery, representing the modified group. The mean age in this group was 39.28 years, with a standard deviation of 17.00 years.

No statistically significant differences were observed in the general data between the two groups (all *P *> 0.05).

It's worth noting that all patients in both groups presented complaints of fecal incontinence (refer to Table 1).

**Table 1:** Baseline characteristics of the study population.

Characteristics	Traditional group *N* = 68	Modified group *N* = 65	*P* *
Age, years	40.87 ± 18.96	39.277 ± 17.00	0.61
Gender			0.56
Male	57.35	52.31	
Female	13.24	47.69	
Education level (%)			0.62
<High school	22.05	21.54	
High school	29.41	36.92	
University and above	48.53	41.54	
Marital status (%)			0.254
Yes	64.71	73.85	
No	35.30	16.15	
Previous surgery (%)			0.913
Yes	14.71	15.38	
No	85.29	84.62	
Anaesthetic risk (%)			0.722
ASA I	1.47	1.54	
ASA II	64.71	44.62	
ASA III	29.41	36.92	
ASA IV	4.41	1.54	
Duration of prolapse, years	38.12 ± 18.60	35.45 ± 15.74	0.372
BMI (kg/m^2)^	23.71 ± 4.03	25.30 ± 5.19	0.051
Maximum prolapse length in squat position (cm)	9.46 ± 3.51	8.97 ± 3.21	0.407
Time of the first postoperative defecation period, days	3.62 ± 1.51	6.60 ± 1.76	<0.001
Postoperative frequency of defecation	5.1 ± 2.81	4.03 ± 2.33	0.018

ASA, American Society of Anesthesiologists; BMI, body mass index.

* *P*^ ^< 0.05.

### Surgical details and treatment outcome

No intraoperative deaths or complications were observed in either group. However, during the mean (12.5 ± 2.41) months of postoperative follow-up, a statistically significant difference in the incidence of postoperative complications emerged between the conventional and modified groups (*P *< 0.001). Specifically, the traditional group had a significantly higher incidence of anastomotic dehiscence (13.24%) compared to the modified group (0, *P *= 0.003). Nevertheless, no significant differences were noted between the two groups in terms of complications such as death, anastomotic hemorrhage, incomplete intestinal obstruction, and anal stenosis (all *P *> 0.05, see Table 2).

**Table 2:** Surgical details and treatment outcome.

	Traditional group *N* = 68	Modified group *N* = 65	*P*
The duration of surgery, min	139.53 ± 24.44	169.34 ± 26.45	<0.001
Intraoperative hemorrhage, ml	27.522 ± 9.21	16.66 ± 4.56	<0.001
Hospitalization time, days	12.82 ± 3.78	15.14 ± 2.40	<0.001
Mortality, %	2.94	0	0.496
Postoperative complications			
Anastomotic dehiscence, %	13.24	0	0.003
Anastomotic hemorrhage, %	17.65	6.15	0.077
Incomplete intestinal obstruction, %	2.94	0	0.496
Anal stenosis, %	4.41	0	0.245
Postoperative recurrence, %	26.47	1.54	<0.001
The time to recurrence, months	8.39 ± 4.28	13 ± 0.00	0.302

During the follow-up period, postoperative recurrence was observed in 26.47% of the patients in the conventional group (*N* = 18), which was significantly higher than the 1.54% recurrence rate in the modified group (*N* = 1) (*P *< 0.001). However, there was no significant difference in the time to recurrence between the two groups (traditional group: (8.39 ± 4.28) months vs. (13 ± 0.00) months in the modified group) (*P *= 0.302, see Table 2).

The results presented in Table 3 indicated improvements in anal resting pressure, squeezing pressure, and Wexner anal incontinence scores both before and after surgery in both groups. In the traditional group, anal resting pressure and squeezing pressure increased from preoperative values of (31.64 ± 1.51) mmHg and (60.68 ± 2.21) mmHg to (32.50 ± 1.78) mmHg and (62.85 ± 2.30) mmHg, respectively, while in the modified group, they increased from (31.67 ± 1.67) mmHg and (61.14 ± 1.91) mmHg to (33.24 ± 2.06) mmHg and (64.78 ± 1.55) mmHg (all *P *< 0.001). Furthermore, Wexner anal incontinence scores decreased from preoperative levels of (4.81 ± 1.79) and (4.25 ± 2.08) to (3.69 ± 1.58) and (2.69 ± 1.65) in the conventional and modified groups, respectively (all *P *< 0.001).

**Table 3:** Sphincter function and Wexner anal incontinence score before and after surgery in two groups.

	Traditional group *N* = 68			Modified group *N* = 65		
	Before the surgery	After the surgery	*P*	Before the surgery	After the surgery	*P*
Resting pressure (cm H_2_O)	31.64 ± 1.51	32.50 ± 1.78	<0.001	31.67 ± 1.67	33.24 ± 2.06	<0.001
Squeeze pressure (cm H_2_O)	60.68 ± 2.21	62.85 ± 2.30	<0.001	61.14 ± 1.91	64.78 ± 1.55	<0.001
Wexner anal incontinence score	4.81 ± 1.79	3.69 ± 1.58	<0.001	4.25 ± 2.08	2.69 ± 1.65	<0.001

There were statistical differences between the two groups in terms of surgical procedures and perioperative outcomes, such as operative time, intraoperative bleeding, and hospitalization days, as shown in Table 2.

## Discussion

Complete rectal prolapse (CRP), a pelvic floor disorder, often arises from chronic diarrhea in infancy, childbirth, neurological injury, or connective tissue disorders (11). This condition typically follows a protracted course, and affected individuals often present with comorbidities. The Altemeier procedure, a representative transperineal surgical approach, is applicable to a broad spectrum of patients but is challenged by recurrence rates and complications, such as anastomotic issues (12, 13).

In this study, we have elucidated a novel anatomical defect in CRP and subsequently tailored the conventional Altemeier procedure to address it (Altemeier procedure combined with Sigmoido-rectal intussusception anastomosis). Our investigation encompassed the follow-up and examination of clinical outcomes, including recurrence, complications, and anal function, in two distinct patient groups. These groups underwent either the conventional Altemeier surgery (comprising 68 patients) or the Altemeier surgery combined with Sigmoido-rectal intussusception anastomosis (comprising 65 patients).

The findings of this study have revealed that patients in the modified group exhibited a significantly lower incidence of postoperative anastomotic dehiscence and a reduced recurrence rate compared to the traditional procedure. Moreover, both the traditional and modified groups demonstrated improved anal function in the short-term follow-up, with the modified group displaying a more favorable enhancement in anal control function.

The recurrence rate has been a significant concern with Altemeier surgery. In a retrospective analysis conducted by Chun et al. (10), which included 109 patients who underwent Altemeier surgery for rectal prolapse, a postoperative recurrence rate of 20.6% was observed. This rate is quite similar to the recurrence rate of conventional Altemeier surgery in our study (26.47%). Interestingly, Cirocco WC (6) did not report any cases of recurrence following Altemeier surgery during their 13-month follow-up of patients with CRP. We believe that the substantial variation in recurrence rates may be attributed to the surgeon's proficiency in performing the Altemeier procedure and the duration of postoperative follow-up.

Additionally, Michal Mik et al. compared postoperative recurrence rates between transperineal and transabdominal surgeries and found no statistically significant difference in recurrence rates between the two groups. In our present study, the same surgeon performed operations on both groups of patients, and the mean follow-up time was (12.5 ± 2.41) months. Notably, postoperative recurrence was significantly lower in the modified group compared to the conventional group. Therefore, the Altemeier procedure combined with Sigmoido-rectal intussusception anastomosis proves effective in reducing postoperative recurrence rates.

Colorectal surgeons at the University of Minnesota have observed that most recurrences after perineal rectosigmoid resection occur within 3 years (7). Our study results align with this observation, as the mean postoperative recurrence time was (8.39 ± 4.28) months in the conventional group and (13 ± 0.00) months in the modified group. These findings not only support the existing literature but also confirm the efficacy of Altemeier's procedure combined with sleeve intestinal anastomosis in delaying the time to recurrence in comparison to the conventional procedure.

However, it's important to note that the relatively small sample size of this study and the absence of long-term follow-up results beyond 10 years render it challenging to conclusively determine whether the modified Altemeier procedure can significantly reduce or entirely eliminate postoperative recurrence. A more precise recurrence rate would require further validation through multicenter studies, larger sample sizes, and extended long-term follow-up.

The hallmark of the Altemeier procedure lies in the intestinal anastomosis performed between the rectum and sigmoid colon. However, this anastomosis can also be a source of anastomotic complications in this procedure. In this study, complications related to the anastomosis were observed in both groups, with a notably higher complication rate in the traditional group compared to the modified group. Nevertheless, a statistically significant difference between the two groups was observed only in the case of anastomotic dehiscence (*P* = 0.003). It's worth noting that this limited variation may be attributed to the relatively small sample size utilized in this study.

Furthermore, it's essential to highlight that the time to postoperative defecation was significantly prolonged in the modified group [(6.60 ± 1.76) days] compared to the conventional group [(3.62 ± 1.51) days, *P* < 0.001]. Delaying the timing of the initial postoperative bowel movement and defecation could potentially contribute to maintaining a relatively clean environment conducive to anastomotic healing, which may serve to reduce the occurrence of complications (14).

Altomare DF et al. reported that as many as 70% of patients with CRP experience varying degrees of fecal incontinence (15). Hence, the benchmark for a successful CRP treatment entails not only achieving low recurrence and complication rates but also the restoration of normal anorectal function.

The findings of the current study revealed a significant improvement in postoperative anal incontinence, as indicated by the Wexner score, and in anal manometry for patients in both the conventional and modified groups (all *P* < 0.001). These improvements align with the results of previous studies. However, it's noteworthy that the enhancement in anal function, both in terms of anal manometry and anal incontinence as measured by the Wexner score, was more pronounced in the modified group compared to the traditional group.

We hypothesize that the primary cause of fecal incontinence in patients with total rectal prolapse may be mechanical compression of the anal sphincter due to prolapse, with a hole shape Anal representing a secondary change. This perspective is consistent with the findings of Park et al. (16). Additionally, an alternative theory suggests that fecal incontinence results from perianal neuromuscular defects or diminished pubic nerve sensitivity. Further investigations will be necessary to validate this hypothesis.

To the best of our knowledge, this is the inaugural clinical study to identify an additional anatomical defect in CRP and subsequently refine the traditional Altemeier procedure based on this revelation. The Altemeier procedure combined with Sigmoido-rectal intussusception anastomosis introduces innovative modifications distinct from the conventional Altemeier procedure. On one hand, it opts for a lower pre-excision line, involving the preservation of a more substantial segment of the muscular propria during the initial stages of the procedure. This innovative approach bears some resemblance to a portion of the Delorme procedure (17).

On the other hand, the Altemeier procedure combined with Sigmoido-rectal intussusception anastomosis exclusively conserves the distal rectal musculature over a span of approximately 2–3 cm, providing a foundation for the subsequent double-layer anastomosis. The paramount innovation in this study, however, lies in the Sigmoido-rectal intussusception anastomosis. Firstly, this intussusception anastomosis forms a double-layer muscular structure within the local intestinal wall, significantly enhancing its ability to withstand abdominal pressure. Secondly, it eliminates the abrupt change in thickness of the local intestinal diameter under the reconstructed pelvic floor peritoneal reflexes, thereby averting the re-entry of the sigmoid colon and effectively diminishing the postoperative recurrence rate associated with traditional Altemeier surgery. Moreover, this anastomosis is embedded within the rectus muscle sheath, affording protection and enhancing its tension resistance, consequently reducing local complications like anastomotic hemorrhage, pelvic abscess, and anastomotic dehiscence (18).

Lastly, this anastomosis not only preserves the normal elasticity and compliance of the rectal wall but also further narrows the terminal intestinal lumen and tightens the anal cushion, which contributes to the restoration of anorectal function. Nevertheless, it's crucial to emphasize that moderate tension is imperative for rectal overlay bowel anastomosis, maintaining a 0.6 cm gap between each suture, as excessive tension can lead to anal stenosis or even ischemic necrosis.

With the advent of laparoscopy, transabdominal surgery has gained widespread popularity as a treatment option for CRP. Nevertheless, we continue to favor the perineal approach for several reasons. This approach allows for surgical revision in cases of treatment failure (19) and is applicable to a diverse range of morbidities.

## Conclusion

Altemeier surgery, being one of the perineal surgical approaches, has encountered limitations associated with a high recurrence rate, which has impeded the widespread adoption of this procedure. However, the innovative “Sigmoido-rectal Intussusception Anastomosis in the Altemeier Procedure” provides a new way of thinking about the choice of surgical procedures for patients with complete rectal prolapse. However, the follow-up period of this article was only (12.5 ± 2.41) months, and a longer follow-up period is needed in the future to confirm the reasonableness of the innovative surgical procedure.

It is imperative to acknowledge that the present study is retrospective in nature and conducted at only three research centers. To establish the clinical efficacy of the modified Altemeier procedure conclusively, further research is warranted, encompassing larger sample sizes, multicenter collaboration, extended follow-up periods, and prospective study designs.

## Data Availability

The original contributions presented in the study are included in the article/Supplementary Material, further inquiries can be directed to the corresponding author.
